# A noninvasive model to predict liver histology for antiviral therapy decision in chronic hepatitis B with alanine aminotransferase < 2 upper limit of normal

**DOI:** 10.1186/s12876-020-01576-6

**Published:** 2021-01-06

**Authors:** Shanshan Chen, Haijun Huang, Wei Huang

**Affiliations:** 1 Department of Infectious Disease, Zhejiang Provincial People’s Hospital, Hangzhou, 310014 Zhejiang China; 2 Graduate School of Clinical Medicine, Bengbu Medical College, BengbuAnhui, 233000 China; 3 Department of Digestive Disease, Zhejiang Provincial People’s Hospital, Hangzhou, 310014 Zhejiang China

**Keywords:** Hepatitis B virus, Liver biopsy, Anti-hepatitis B virus core antibody (anti-HBC), Noninvasive model

## Abstract

**Background:**

At present, most assessments of liver fibrosis staging mainly focus on non-invasive diagnostic methods. This study aims to construct a noninvasive model to predict liver histology for antiviral therapy in chronic hepatitis B (CHB) with alanine aminotransferase (ALT) < 2 times upper limit of normal (ULN).

**Methods:**

We retrospectively analyzed 577 patients with CHB who received liver biopsy and whose ALT was less than 2 ULN. Then they were randomly divided into a training group and a validation group. Through logistic regression analysis, a novel predictive model was constructed in the training group to predict significant changes in liver histology [necro-inflammatory activity grade (G) ≥ 2 or fibrosis stage (S) ≥ 2] and then validated in the validation group.

**Results:**

If liver biopsy showed moderate or severe inflammation or significant fibrosis, antiviral treatment was recommended. Aspartate aminotransferase (AST), anti-hepatitis B virus core antibody (anti-HBC) and glutamine transpeptidase (GGT) were identified as independent predictors for antiviral therapy, with area under the ROC curve (AUROC) of 0.649, 0.647 and 0.616, respectively. Our novel model index, which combined AST, anti- HBC and GGT with AUROC of 0.700 and 0.742 in training set and validation set.

**Conclusions:**

This study established a noninvasive model to predict liver histology for antiviral treatment decision in patients with CHB with ALT < 2 ULN, which can reduce the clinical needs of liver biopsy.

## Background

Hepatitis B virus (HBV) infection is still a major public health problem in the world. It is one of the most serious and common health problems, affecting more than 2 billion people [1]. Once HBV chronic infection, the covalently closed circular DNA (ccc-DNA) was difficult to be removed from hepatocytes.

Studies have shown that HBV replication and subsequent immune-mediated liver damage are the main key factors for disease progression. The inevitable progression of HBV infection to persistent or intermittent liver inflammation and fibrosis greatly increases the risk of progression to cirrhosis, Hepatocellular carcinoma (HCC) and end-stage liver disease [2]. Early and timely antiviral treatment can reverse liver fibrosis and early cirrhosis, thereby reducing the incidence of HCC [3–5].Therefore, the main purpose of chronic hepatitis B virus (CHB) treatment is to inhibit HBV replication, reduce the occurrence of liver decompensation and prevent the progress of disease as soon as possible [1]. The recent American Association for the Liver Diseases (AASLD) guidelines recommend antiviral therapy when alanine aminotransferase (ALT) ≥ 2 upper limit of normal (ULN), moderate or severe inflammation or significant fibrosis and HBV-DNA elevation [6]. European Association for the Study of the Liver (EASL) guidelines recommend that patients with ALT > ULN, moderate or severe inflammation or obvious fibrosis and HBV-DNA elevation should receive antiviral treatment [7].

The level of ALT is able to reflect the severity of liver injury and is an important biochemical index to evaluate liver injury. However, due to its effectiveness was limited, not all patients with HBV infection will continue to increase of ALT level [8, 9]. About one fifth of patients with CHB with normal ALT level may have significant liver fibrosis [10]. Patients with high ALT level are more likely to have liver inflammation or fibrosis, but those with long-term ALT level lower than 2 ULN value of HBV infection, liver biopsy is recommended. However, due to the invasive nature of liver biopsy with serious complications, short-term non-repeatability, sampling errors and differences between observer and within the observer, the examination was rejected by some patients [11, 12]. Therefore, more and more attention has been paid to the prediction of liver histology by noninvasive diagnostic methods.

In this study, we aimed to constructed a noninvasive model based on serum markers to accurately identify moderate and severe inflammation or significant fibrosis related to CHB, which is helpful to the decision-making of antiviral treatment in patients with chronic hepatitis B with ALT < 2 ULN and to reduce the need for liver biopsy as much as possible.

## Methods

This study retrospectively analyzed 577 patients with CHB who underwent liver biopsy from October 2008 to November 2019 in the Department of infectious diseases of Zhejiang Provincial People's Hospital. According to the guidelines of Helsinki declaration in 1975 and the approval of the local research and ethics committee of Zhejiang Provincial People's Hospital, all patients were given written informed consent for analysis. HBV infection was defined as HBsAg positive for at least 6 months [13]. The inclusion criteria include: the diagnosis of CHB infection, the level of ALT less than 2 ULN (ULN = 40U/L), detectable HBV-DNA with a level > 10^2^ IU/ml. Exclusion criteria include: such as hepatitis C virus (HCV), hepatitis D virus (HDV), hepatitis E virus or human immunodeficiency virus (HIV) Co-infection and other causes of liver disease, alcoholic liver disease, decompensated cirrhosis, HCC, nonalcoholic fatty liver disease (NAFLD). Autoimmune liver disease, insufficient liver biopsy samples and incomplete clinical data.

### Laboratory tests

Before liver biopsy, we collected the patient's demographic (age and gender) and clinical laboratory parameters, including serological, biochemical and virus indicators, white blood cell (WBC), platelet (PLT), albumin (ALB), globulin (GLB), total bilirubin (TBIL), alanine aminotransferase (ALT), aspartate aminotransferase (AST), glutamyl transpeptidase (GGT), alkaline phosphatase (ALP). The serum HBV-DNA load was assessed by a real-time polymerase chain reaction System (ABI7300, Applied Biosystems, Foster City, CA, USA). HBsAg and HBeAg were determined by automatic analyzer.

### Liver biopsy

All patients received ultrasound-guided percutaneous liver biopsy. Liver biopsy was performed using an 18G biopsy needle. The biopsy samples were fixed with formalin and embedded in paraffin. Hematoxylin and eosin (HE), Masson trichrome staining and reticular fiber staining were used to evaluate the stages of inflammatory necrosis and fibrosis. The liver biopsy samples were at least 1.5 cm in length and contained at least 6 complete portal vein bundles. Necrotizing inflammation (G0-G4) was histologically graded and hepatic fibrosis (S0-S4) was staged according to the Scheuer classification system [14]. Liver fibrosis was considered significant when it spread beyond the portal tract (S2-S4). All sections were independently evaluated by two pathologists, the observation results were processed by kappa consistency test. The two pathologists were not aware of the patient's condition. When the results of the two pathologists were inconsistent, they reevaluated and analyzed the differences and reached a consensus. According to the changes of liver histology, the patients were divided into mild group (G < 2 and S < 2) and significant group (G ≥ 2 or S ≥ 2). According to the related guidelines, patients of the significant group should be given antiviral treatment.

### Statistical analysis

Statistical analyses of the data were performed with SPSS software (version 21.0, SPSS Inc., IBM, Chicago, IL, USA). The quantitative variables were expressed as mean ± standard deviation (SD) or the range of quartiles, then compared with Student’s t-test or non-parametric test (Mann–Whitney). The classification variables are represented by numbers or percentages and compared by chi-square test. The independent risk factors of necrotic inflammation and liver fibrosis were predicted by logistic regression. The area under the AUROC curve was analyzed and evaluated, and the cut-off value was determined by the ROC at the maximum Jordan index. The accuracy of diagnosis was evaluated by calculating sensitivity, specificity, positive predictive value (PPV) and negative predictive value (NPV). Finally, verify in the verification set. A two-tailed value of *P* < 0.05 indicated to statistical significance.

## Results

### Demographic and clinical characteristics of patients

In this study, 577 patients with HBV infection with ALT less than 2 ULN were randomly divided into two groups, including training set (n = 347) and verification set (n = 230). A total of 179 cases (51.5%) in the training set and 113 cases (49.1%) in the verification set were identified as moderate or severe inflammation. A total of 181 cases (52.2%) in the training set and 134 cases (58.2%) in the verification set were identified as obvious fibrosis, and they were identified as requiring antiviral treatment.

The analysis of demographic and clinical laboratory indicators of the population included in the liver biopsy was shown in Table 1. Among all statistical variables, there was no significant difference between training set and verification set.

**Table 1:** Characteristics of patients in the training and validation sets

Variables	Training set (n = 347)	Validation set (n = 230)	*P* value
Age (years)	37.34 ± 9.76	38.62 ± 10.40	0.132
Male gender, n (%)	220 (63.4%)	138 (60%)	0.410
WBC (× 10^9^/ L)	5.80 ± 1.48	5.87 ± 1.68	0.597
PLT (× 10^9^/ L)	187.00 (154.00–214.00)	186.00 (148.00–217.25)	0.882
TBIL (µmol/L)	15.10 (12.30–18.60)	14.30 (11.50–18.50)	0.100
ALB (g/L)	44.90 (42.90–46.90)	44.40 (42.30–45.80)	0.035
GLB (g/L)	29.05 (26.88–32.03)	29.45 (26.65–32.33)	0.799
GGT (U/L)	20.00 (15.00–30.00)	20.50 (15.00–31.25)	0.792
ALP (U/L)	80.00 (65.00–93.00)	79.00 (67.00–96.00)	0.346
ALT (U/L)	33.00 (22.00–50.00)	32.00 (20.00–48.25)	0.213
AST (U/L)	28.00 (23.00–37.00)	28.00 (22.00–36.00)	0.348
HBeAg, Positive N (%)	179 (51.6%)	122 (53.0%)	0.652
Necro-inflammatory active grade G0/G1/G2/G3/G4	4/164/149/30/0	5/112/89/23/1	0.451
Fibrosis stage S0/S1/S2/S3/S4	21/145/122/26/33	12/83/96/14/24	0.492
Anti-HBC (S/CO)	10.27 (8.70–11.55)	10.02 (8.79–11.22)	0.348
HBV-DNA (IU/mL)	69,500.00(2250.00–23,000,000.00)	95,000.00(2200.00–17,000,000.00)	0.972
G ≥ 2, N (%)	179 (51.5%)	113 (49.1%)	
S ≥ 2, N (%)	181 (52.2%)	134 (58.2%)	

*WBC* white blood cell, *PLT* platelet, *TBIL* total bilirubin, *ALB* albumin, *GLB* globulin, *GGT* glutamyl transpeptidase, *ALP* alkaline phosphatase, *ALT* alanine aminotransferase, *AST* aspartate aminotransferase, *Anti-HBC* anti-hepatitis B virus core antibody, *HBeAg* hepatitis B e antigen

### Independent factors related to antiviral treatment decisions and their manifestations

In order to study the independent variables of antiviral treatment decision-making in patients with HBV infection whose ALT is less than 2 ULN, and to identify moderate and severe inflammation or significant fibrosis. We made statistical analysis on demographic and clinical laboratory indicators, and made logistic regression to analyze the work characteristic curve of subjects.

**Fig. 1:**
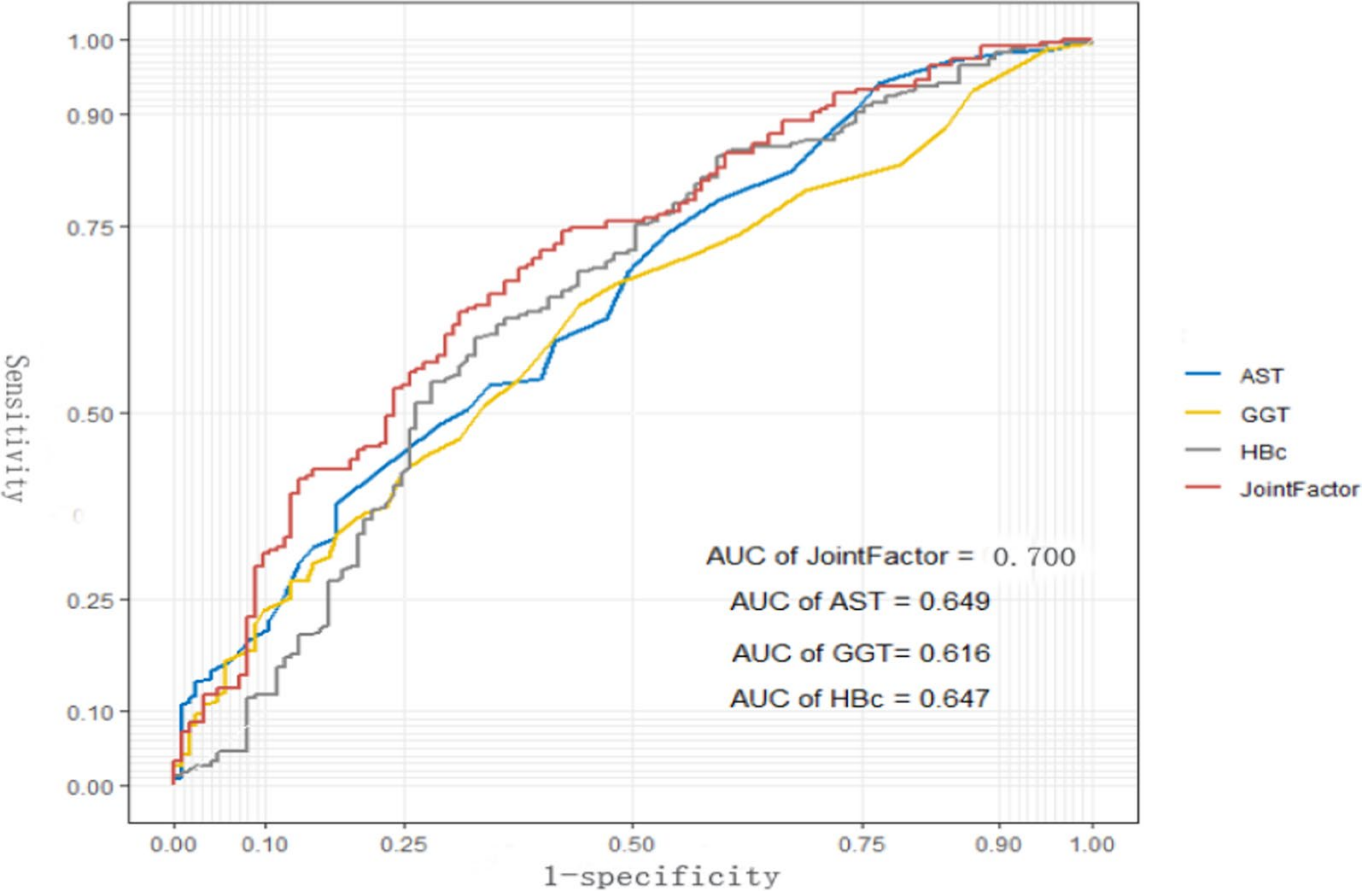
The ROC of independent variables and the AGH model for identifying moderate or severe inflammation or significant fibrosis in the training set. The AUROC of the AGH model was 0.700, which was higher than that for AST (0.649), GGT (0.616) and Anti-HBC (0.647) alone in the training set. AST, aspartate aminotransferase; Anti-HBC, anti-hepatitis B virus core antibody; GGT, glutamyl transpeptidase; AUROC, area under the receiver operating characteristic curve;

As shown in the Fig. 1, the subject operating characteristic curve was analyzed to evaluate the diagnostic performance of the independent variables were used to identify moderate or severe inflammation or significant fibrosis. In the training set, the AUROC of AST, anti HBC and GGT were 0.649, 0.647 and 0.616, respectively.

### Nomogram of the incidence of liver fibrosis

**Fig. 2:**
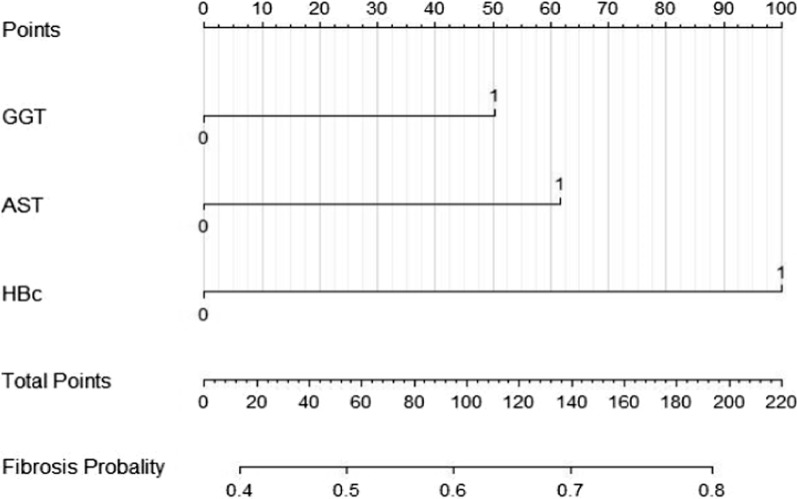
Nomogram of the incidence of liver fibrosis. AST, aspartate aminotransferase; Anti-HBC, anti-hepatitis B virus core antibody; GGT, glutamyl transpeptidase; For clinical use of the model, the total scores would be calculated according to the score of each variable from individual index, and the probability of liver fibrosis could be determined

We used nomogram to predict the incidence of liver fibrosis. The consistency index (C index) was used to determine the prediction accuracy and discrimination ability of nomogram. In our study, AST, GGT and Anti-HBC were considered as variables in the liver fibrosis nomogram (Fig. 2).

### The combination of AST, anti-HBC and GGT showed better diagnostic performance

On the basis of the above independent variables, construct the combination of index and analyze whether the performance of the combination index can be improved through logistic regression. Finally, based on the binary forward stepwise logistic analysis of training set, an AGH model composed of AST, anti HBC and GGT is proposed (Table 2). The publicity of the combined factors was: AGH index = 0.033 * AST (U / L) + 0.016 * GGT (U/L) + 0.193 * anti-HBC (S/CO)—2.745;

**Table 2:** Multivariate logistic regression analysis of independent predictors for significant histological change in training set

Variable	AUC	95% CI	*P* value
AST (U/L)	0.649	0.589–0.709	< 0.001
GGT (U/L)	0.616	0.556–0.676	< 0.001
Anti-HBC (S/CO)	0.647	0.584–0.709	< 0.001
Joint factor	0.700	0.643–0.757	< 0.001

*AST* aspartate aminotransferase, *Anti-HBC* anti-hepatitis B virus core antibody, *GGT* glutamyl transpeptidase, *AUC* area under the curve, *CI* confidence interval

**Fig. 3:**
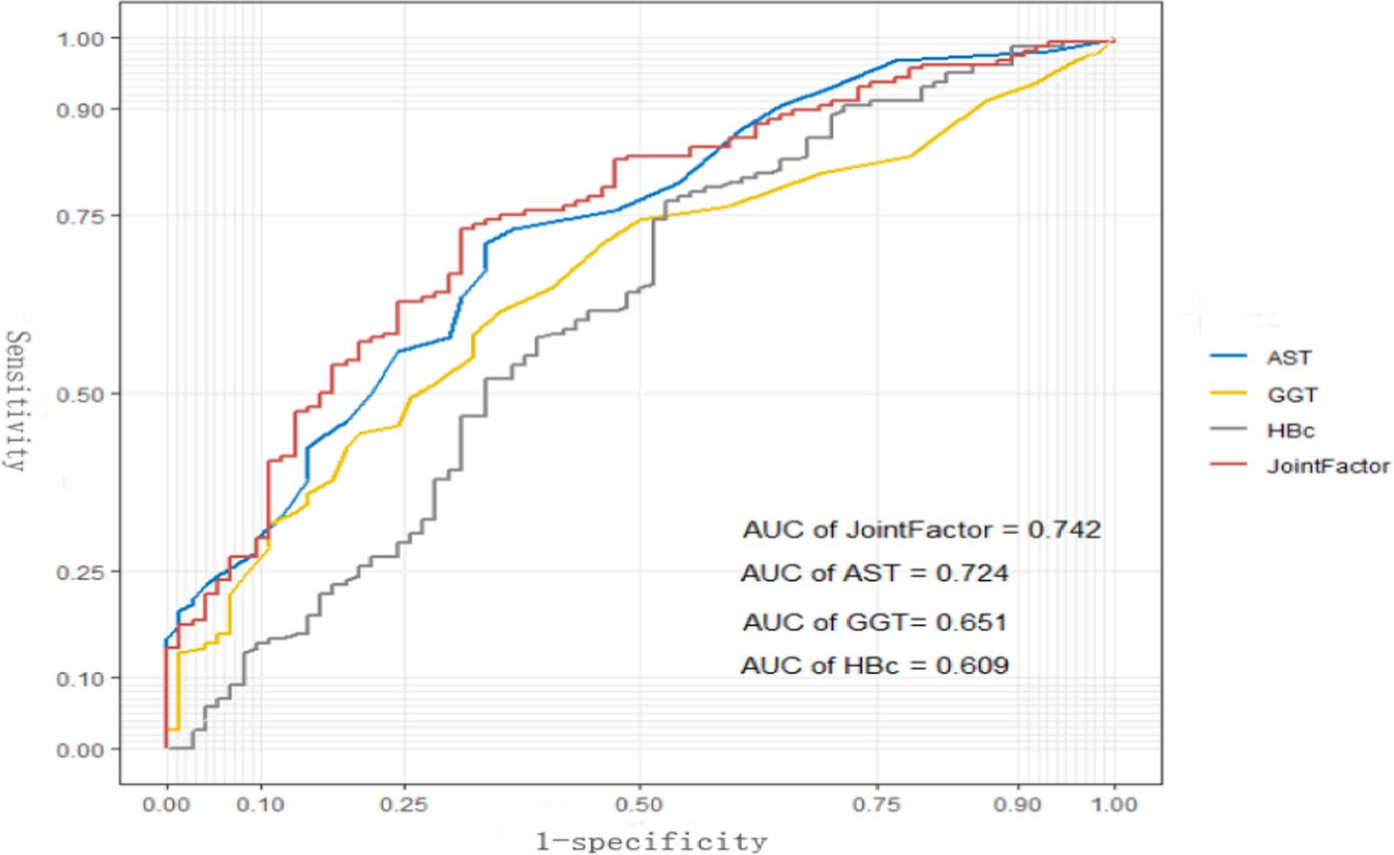
The ROC of independent variables and the AGH model for identifying moderate or severe inflammation or significant fibrosis in the validation set. The AUROC of the AGH model was 0.742, which was higher than that for AST (0.724), GGT (0.651) and Anti-HBC (0.609) alone in the validation set. AST, aspartate aminotransferase; Anti-HBC, anti-hepatitis B virus core antibody; GGT, glutamyl transpeptidase; ROC, the receiver operating characteristic curve;

In the training set, the AUROC of AGH index was 0.700, higher than that independent variables of AST, anti-HBC and GGT. In the validation set, the AGH model also showed good performance and the AUROC of AGH model was 0.742, higher than that independent variables of AST, GGT and Anti-HBC (Fig. 3).

On the basis of ROC analysis, the cut-off value of AGH index was calculated. Selecting a high cut-off value (> 0.215) to determine patients with moderate or severe inflammation or significant fibrosis and need antiviral treatment. Selecting a low cut-off value (< 0.205) to determine patients without moderate or severe inflammation or significant fibrosis and can be followed up regularly. Their diagnostic performance including sensitivity, specificity, positive predictive value and negative predictive value were calculated (Table 3). According to the critical value of AGH index is 0.503, it is suggested that 200 patients should start to antiviral treatment and 38 patients should be followed up regularly.

**Table 3:** Multivariate logistic regression analysis of moderate inflammation or significant fibrosis in the training set and validation set

Patients	AUROC	*P* value	Cut-off values	95% CI	Sensitivity (%)	Specificity (%)
Training set	0.700	< 0.001	0.328	0.643–0.757	0.64	0.688
Validation set	0.742	< 0.001	0.417	0.674–0.810	0.732	0.685

*AUROC* area under the receiver operating characteristic, *CI* confidence interval

AGH model shows that a good performance in training set and verification set, and its AUROC is 0.700 and 0.742 respectively. Finally, 404(70.0%) out of among 577 patients with chronic hepatitis B could avoid liver biopsy according to the critical value.

## Discussion

In this study, we established a noninvasive model to predict liver histology to determine moderate or severe inflammation or significant fibrosis, and to guide the decision-making of antiviral treatment in patients with chronic hepatitis B with ALT < 2 ULN. The results showed that AST, anti-HBC and GGT were independent risk factors for antiviral therapy. The combination of AST, anti- HBC and GGT has better diagnostic performance than single variable.

In patients with chronic hepatitis B, persistent liver inflammation is a major risk factor for the development of cirrhosis, HCC and end-stage liver disease [1, 12]. The latest guidelines suggest that early and timely control of liver inflammation to prevent disease progression to end-stage liver disease is the primary task of liver fibrosis detection [15]. The latest EASL guidelines point out that antiviral therapy should be performed when ALT > ULN [7]. The AASLD guidelines indicate that patients with ALT > ULN but < 2 ULN need to consider the severity of liver histological changes [6]. Therefore, it is very important to evaluate the progress of liver inflammation and fibrosis in the early stage for the treatment and prognosis in the later stage. However, liver biopsy is an invasive operation with related complications, and its clinical application is limited [16]. At present, most assessments of liver fibrosis staging focus on non-invasive diagnostic methods, such as transient elastography (TE). But TE itself has certain limitations, such as TE requires specialized centers, so it is less readily available, limited applicability in obese and ascites patients [17], and the results of TE are also affected by liver inflammation and activity [18]. Therefore, our research is to avoid the clinical need of liver biopsy by establishing noninvasive, repeatable and rapid indicators of antiviral treatment decision-making.

Serum ALT level is one of the main biochemical markers to evaluate liver inflammation. A study in a Korean population showed that some people with high viral load and ALT < 2 ULN had significant liver necrosis inflammation and fibrosis [19]. About 37% of CHB patients with normal or near normal ALT level, but had significant liver histologic changes [20–22]. The results showed that ALT level was not enough to assess the severity of liver injury [22]. Some studies have shown that AST has better diagnostic performance than ALT in predicting hepatic necrotizing inflammation [19]. As an independent factor to predict significant fibrosis, AST level increased with the aggravation of liver fibrosis [23]. Some of the delay clearance in AST was related to ALT level [24], and some of the advanced liver fibrosis was related to mitochondrial damage [25]. Another study suggested that the increase of AST level was related to the advanced fibrosis [26]. Our study shows that AST level can better predict moderate and severe inflammation and significant fibrosis. Therefore, we should be closely monitored and evaluated AST level regularly.

Some studies have suggested that anti-HBC in serum biomarkers is a sensitive marker in the history of chronic HBV infection. After the appearance of HBsAg in the early stage of HBV infection, anti-HBC antibodies will be detected in serum soon [27, 28]. Serum anti-HBC level is closely related to the state of HBsAg and the activity of liver inflammation [28]. In patients with chronic hepatitis B whose ALT level is less than 2 ULN, the serum anti-HBC level has a better diagnostic performance in predicting moderate and severe liver inflammation, even in patients with normal or slightly elevated ALT level [22, 23]. Li et al. founded that the anti-HBC level increased with gradually severity of hepatic inflammation and fibrosis stage. Anti-HBC showed a high diagnostic accuracy for identifying moderate or severe inflammation in all patients and patients with ALT lower than 64 IU/L (AUROC = 0.768 and 0.767, respectively) in a study of 469 treatment-naïve CHB patients [22]. In our study, Anti-HBC is a major predictor of liver fibrosis, which has good diagnostic performance in predicting liver inflammation and necrosis.

In patients with chronic hepatitis B, GGT as an independent variable has a higher diagnostic performance for significant fibrosis [29]. GGT is more stable in predicting liver inflammation than ALT and AST. GGT is an important predictor of noninvasive fibrosis in patients with HBV infection [30–32]. Therefore, GGT is a reliable predictor of liver histological changes in routine laboratory indexes. We should regularly monitor the dynamic changes of this index and give drug treatment when necessary.

The nomogram not only reflects the predicted value of each variable, but also reflects the complex interaction with other variables [33]. Through constructing the model, clinicians can more accurately assess the incidence of liver fibrosis, so as to provide guidance for better monitoring and treatment of patients with chronic hepatitis B.

According to logistic analysis, we set up a new combined model, which combines AST, anti-HBC and GGT. This combined model is simple and easy to use, and has a good effect on the decision-making of antiviral therapy. Through ROC curve analysis, the combined index is 0.700 and 0.742 in training set and verification set respectively, which is higher than AST, anti-HBC and GGT. If AGH index is higher than the high critical value, antiviral therapy is recommended; if AGH index is lower than the low critical value, regular follow-up is recommended; if AGH index is between the low and high critical value, liver biopsy is recommended.

The treatment of chronic hepatitis B was mainly based on serum HBV-DNA level, serum ALT level and hepatic histological severity [34]. However, in our study, single-factor and multi-factor logistic regression analysis showed that HBV-DNA level was not an independent factor influencing antiviral treatment. Therefore, the variable HBV-DNA level was not included in AGH model. Similarly, through logistic regression analysis, we found that HBeAg was not an independent factor in antiviral treatment decision. Previous studies have shown that HBeAg-positive and HBeAg-negative chronic hepatitis B patients have different factors related to liver histology [35]. AST, HBsAg, platelet (PLT) and albumin (ALB) were independent factors in HBeAg-positive patients [35]. Age, AST, HBV-DNA and PLT were independent factors in HBeAg-negative patients [36].

Our research has its own advantages. AGH model is a new index, which is composed of clinical routine laboratory tests and easy to obtain. It is suitable for most patients with chronic hepatitis B virus infection as well as be used to predict liver histology to avoid the need of liver biopsy. Our model used only serum markers which made it suitable for most medical facilities. We further evaluated the performance of other non-invasive models in antiviral treatment decision-making. As other models like Forn’s model, AST/ALT ratio (AAR), AST to PLT ratio Index (APRI), Fibrosis Index, FIB-4, Hui’s model, Goteborg University Cirrhosis Index (GUCI) and Zeng’s model [37–44]. Generally, the diagnostic performance of most non-invasive models is lower than AGH model, but not as good as that of APRI and FIB-4, probably because of the different choices of participants.

This study also has some defects in this model. Firstly, the number of patients included is small, the clinical application should be included in a large number of patients for multicenter cohort study. Secondly, we constructed the model in the training set and randomly select another group in the same center to verify, but we insist that the credibility of the model can be enhanced by the validation in the multicenter study.

## Conclusions

Our study shows that AST, Anti-HBC and GGT are independent variables in the decision-making of antiviral therapy for chronic hepatitis B. AGH model which is a new combined index, has a good diagnosis performance. It can provide a noninvasive prediction model for CHB patients to choose antiviral therapy, liver biopsy or regular follow-up, and help to reduce the clinical needs of liver biopsy.

## Data Availability

Liver biopsy and pathology reports from December 2008 to November 2019 were obtained from the electronic medica records of Zhejiang Province People’s Hospital (Hangzhou, China). The datasets generated and/or analyzed during this study are not publicly available given our commitment to patient privacy rights. However, anonymous data may be requested from the corresponding author for valid use.

## Abbreviations

CHB: Chronic hepatitis B
HBV: Hepatitis B virus
WBC: White blood cell
PLT: Platelet
TBIL: Total bilirubin
ALB: Albumin
GLB: Globulin
GGT: Glutamyl transpeptidase
ALP: Alkaline phosphatase
ALT: Alanine aminotransferase
AST: Aspartate aminotransferase
Anti-HBC: Anti-hepatitis B virus core antibody
HBeAg: Hepatitis B e antigen
ULN: Upper limit of normal
AUC: Area under the curve
CI: Confidence interval
AUROC: Area under the receiver operating characteristic
