# Correction to: A noninvasive model to predict liver histology for antiviral therapy decision in chronic hepatitis B with alanine aminotransferase < 2 upper limit of normal

**DOI:** 10.1186/s12876-021-01649-0

**Published:** 2021-03-05

**Authors:** Shanshan Chen, Haijun Huang, Wei Huang

**Affiliations:** 1 Department of Infectious Disease, Zhejiang Provincial People’s Hospital, Hangzhou, 310014 Zhejiang China; 2 Graduate School of Clinical Medicine, Bengbu Medical College, Bengbu, 233000 Anhui China; 3 Department of Digestive Disease, Zhejiang Provincial People’s Hospital, Hangzhou, 310014 Zhejiang China

## Correction to: BMC Gastroenterol (2021) 21:4 https://doi.org/10.1186/s12876-020-01576-6

Following publication of the original article [1], the authors identified incorrect corresponding author mentioned. Correct corresponding author is Haijun Huang.
